# Magnetic-Electrospinning Synthesis of γ-Fe_2_O_3_ Nanoparticle–Embedded Flexible Nanofibrous Films for Electromagnetic Shielding

**DOI:** 10.3390/polym12030695

**Published:** 2020-03-20

**Authors:** Jie Zheng, Bin Sun, Xiao-Xiong Wang, Ze-Xing Cai, Xin Ning, Saad M. Alshehri, Tansir Ahamad, Xing-Tao Xu, Yusuke Yamauchi, Yun-Ze Long

**Affiliations:** 1 Industrial Research Institute of Nonwovens & Technical Textiles, College of Textiles & Clothing, Qingdao University, Qingdao 266071, China; zhengjie2009123@126.com (J.Z.); xning_irintt@163.com (X.N.); 2 Collaborative Innovation Center for Nanomaterials & Devices, College of Physics, Qingdao University, Qingdao 266071, China; wangxiaoxiong69@163.com; 3 International Center for Materials Nanoarchitectonics (WPI-MANA) and International Center for Young Scientists (ICYS), National Institute for Materials Science (NIMS), 1-1 Namiki, Tsukuba, Ibaraki 305-0044, Japan; cai.zexing@kochi-tech.ac.jp; 4 Department of Chemistry, College of Science, King Saud University, Riyadh 11451, Saudi Arabia; alshehri@ksu.edu.sa (S.M.A.); tahamed@ksu.edu.sa (T.A.); 5 School of Chemical Engineering and Australian Institute for Bioengineering and Nanotechnology (AIBN), The University of Queensland, Brisbane, QLD 4072, Australia; y.yamauchi@uq.edu.au; 6 Department of Plant & Environmental New Resources, Kyung Hee University, 1732 Deogyeong-daero, Giheung-gu, Yongin-si, Gyeonggi-do 446-701, Korea

**Keywords:** nanofibrous magnetic films, magnetic field, magnetic-electrospinning, electromagnetic shielding, flexibility

## Abstract

The exploration of a new family of flexible and high-performance electromagnetic shielding materials is of great significance to the next generation of intelligent electronic products. In this paper, we report a simple magnetic-electrospinning (MES) method for the preparation of a magnetic flexible film, γ-Fe_2_O_3_ nanoparticle-embedded polymeric nanofibers. By introducing the extra magnetic field force on γ-Fe_2_O_3_ nanoparticles within composite fibers, the critical voltage for spinning has been reduced, along with decreased fiber diameters. The MES fibers showed increased strength for the magnetic field alignment of the micro magnets, and the attraction between them assisted the increase in fiber strength. The MES fibers show modifications of the magnetic properties and electrical conductivity, thus leading to better electromagnetic shielding performance.

## 1. Introduction

The nanofibrous film is the focus of a comprehensive area of research, particularly in material science and device engineering. The exploitation of new applications of nanofibrous film and its derived nanostructures is undoubtedly one of the hottest topics in the material field over the years [1,2,3,4]. Many breakthrough achievements have been made in applications including tissue engineering, filtration, nano-sensors and nano-electronics, composite reinforcement, energy harvesting and storage, electromagnetic interference shielding, and water treatment [5,6,7,8,9]. For example, Liu et al. reported on a high-performance water desalination electrode based on graphene-bonded nanofibers [10]. Miao et al. developed an electrospinning polyimide nanofiber-based nonwoven Li-battery separator and promoted the development of electrospinning nanofiber-based battery separators [11]. Lin et al. proposed an electrospinning construction of a polyaniline nanofiber humidity sensor [12]. Recently, more and more scientific research has focused on electromagnetic interference (EMI) shielding materials to counter the great trouble EMI causes electronic communications and highly sensitive electronic equipment [13,14]. EMI-shielding materials work by converting electromagnetic radiation into thermal energy through the interaction of electromagnetic waves with the electromagnetic dipoles of charge carriers (electrons or holes) on the surface and inside the material. Therefore, the composition, conductivity, and thickness of EMI-shielding materials are key factors directly affecting EMI-shielding performance [13]. Metals and metalized materials are well known as the best EMI-shielding materials for high conductivity. However, metals with poor flexibility and heavy weight have limited practical application in smart portable and wearable electronics. Therefore, preparing a practical, flexible, lightweight, and efficient EMI-shielding material is a huge challenge that has attracted the interest of many researchers at home and abroad [15,16,17,18,19,20,21]. In addition to the progress in this area, the development of a new family of flexible electromagnetic shielding materials based on nanofibrous films is also of great interest [13,14,22,23,24,25,26,27,28,29]. For example, Ji et al. reported on a highly flexible porous electrospinning crosslinked polyacrylonitrile nanofiber decorated with metal (Ag, Cu, Ni) nanoparticles that exhibited an excellent EMI-shielding performance [13]. Erdem et al. analyzed the EMI-shielding effectiveness and mechanical properties of sputter-coated electrospinning nanofibers [14]. Blachowicz reviewed recent developments in electrospinning magnetic nanofibers and application, specifically discussing EMI-shielding [29]. Generally, these flexible electromagnetic shielding materials could be prepared by directly combining polymeric precursors with magnetic nanofillers. However, the aggregation of magnetic nanofillers during the synthesis process usually limits the performance of prepared magnetic polymeric films. Thus, it is highly advantageous to develop a simple and effective method for the precise preparation of magnetic flexible polymeric fibrous films.

Electrospinning is undoubtedly the most widely used method for preparing nanofibrous films [30,31]. As-spun fibers with fascinating properties, such as large surface areas, high length-to-diameter ratios, flexible surface functionality, tunable surface morphologies, and superior mechanical performances, have recently attracted much attention and have a wide array of potential applications, including for thermal management [32,33], optoelectronics [34,35], sensors [36,37,38], catalysis [39,40], energy storage [41,42], tissue engineering [43], and drug delivery [44,45]. The increasing demand in recent years for the precise preparation of nanofibrous films has accelerated the development of new-concept electrospinning techniques. For example, a double-spinning method was reported that combined electrospinning and centrifuge spinning to fabricate aligned superfine fibrous arrays [46]. A magneto-mechanical drawing method was proposed to fabricate continuous microfibers [47]. Near-field electrospinning (NFES) is a new electrospinning technique that has been developed for the precise control of fiber deposition. Since it was first proposed in 2006 [48], NFES has been the subject of enormous interest by researchers worldwide as it only needs a small electric field to produce continuous fibers with diameters smaller than those with conventional electrospinning [49]. However, the small spinning distance of NFES, decreasing from tens of centimeters to a few microns, would lead to insufficient space for fibers to stretch and split further, thus resulting in the production of thicker fibers. Improving the spinning voltage could reduce the fiber diameter to some extent, but this dangerous operation would cause higher energy consumption and more environmental issues. It is well known that the introduction of an external magnetic force during the electrospinning process could effectively reduce the spinning voltage and alter the morphology, diameter, and arrangement of electrospinning fibers [50,51,52]. However, this concept has not been adopted for NFES. Considering that the short spinning distance of NFES coincides with the effective distance of the magnetic force, the combination of the external magnetic field with the NFES process should be feasible.

In this work, a new magnetic-electrospinning (MES) method—magnetic field-auxiliary NFES—has been developed and applied to the preparation of flexible magnetic fibrous films of a γ-Fe_2_O_3_-embedded polyvinylpyrrolidone (PVP) nanofibrous system. Introducing an external magnetic force into the NFES process narrows the fibrous diameter and decreases the critical voltage. On the other hand, the aggregation of magnetic γ-Fe_2_O_3_ particles is eased to some extent, giving rise to the relatively uniform distribution of γ-Fe_2_O_3_ nanoparticles in the PVP nanofibers. When applied to electromagnetic shielding materials, the flexible magnetic fibrous film shows good electromagnetic shielding effectiveness, suggesting the potential of such flexible magnetic fibrous films for practical application.

## 2. Experimental Section

### 2.1. Materials and Preparation of the Spinning Solution

Polyvinyl pyrrolidone (PVP; *M_w_* ~10000), purchased from Aladdin, Ltd. (Shanghai, China), was used as a solute. Ethanol purchased from Yantai Sanhe, Ltd., was used as a solution. γ-Fe_2_O_3_ nanoparticles with average diameters of 20 nm were purchased from Hefei Kejin, Ltd (Heifei, China). Typically, 5 g of PVP was dissolved in 15 g of ethanol and stirred for 2 h at room temperature to make a uniform and stable precursor solution with 25 wt % PVP. Then, γ-Fe_2_O_3_ nanoparticles with a weight of 0.202, 0.408, and 0.619 g were dispersed into the above precursor solution, respectively, under ultrasonication at 40 KHz for 30 minutes in an ice water bath. After that, clear spinning solution was obtained, and the mass ratios of γ-Fe_2_O_3_ were 1, 2, and 3 wt %, respectively.

### 2.2. Magnetic-Electrospinning (MES) Setup

**Figure 1:**
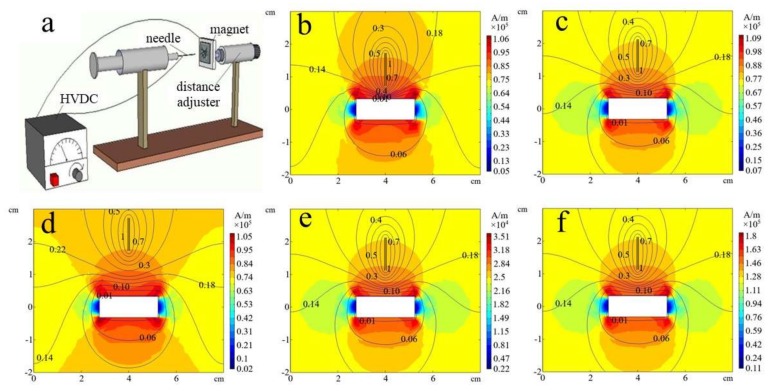
(**a**) A schematic illustration of the magnetic-electrospinning (MES) setup. Theoretical simulations of electric-magnetic fields (**b**–**f**) in the MES share the same voltage (2 Kv) but different distances (4, 8, and 14 cm) and magnetic field strengths (30, 93, and 154 mT); (**b**) 4 cm and 93 mT; (**c**) 8 cm and 93 mT; (**d**) 14 cm and 93 mT; (**e**) 8 cm and 30 mT; and (**f**) 8 cm and 154 mT. The X-axis and the left Y-axis of (**b**–**f**) indicate the distance in cm, and the color bar of the right Y-axis indicates the magnetic field strength (*H*) in A/m.

The electrospinning of nanofibers was performed on a magnetic field-auxiliary electrospinning setup (named magnetic-electrospinning (MES)), which is schematically illustrated in Figure 1a. A high-voltage DC (HVDC) power supply (DW-P403-1ACCC, Tianjin Dongwen (Tianjin, China)) was used to supply a high-voltage electrostatic field for MES. A medical syringe was used to accommodate the spinning solution, and the attached metal needle tip (with an outer diameter of 0.72 mm and inner diameter of 0.42 mm) connected to the positive electrode of the power supply was applied as the spinning tip. A piece of flat aluminum foil connected to the negative electrode of the power supply with a distance to the needle tip of less than 14 mm was used as the collector for electrospinning fibers and was stuck to the surface of a permanent magnet. One of three permanent magnets (purchased from Dongguan Yikai, Ltd.(Dongguan, China), of the same size (2.47 × 1.62 × 0.64 cm = 2.56 cm^3^)) was used by itself each time to generate a magnetic field with different surface magnetic field strengths (30, 93, and 154 mT). The spinning distance was controlled by fixing the permanent magnet and the grounded aluminum foil together on a distance adjuster. To avoid the dropping of droplets hanging vertically from the needle and the covering of the spun nanofibers, the needle and the collector were placed on the same horizontal line so that the droplets could move horizontally and parabolically under the action of gravity, and they were separated from the electrospinning nanofibers, as discussed in detail in our previously published paper [53]. The spinning solution reaches the needle tip through the syringe (the spinning solution throughput is nearly 2 ml/h). When the HVDC power supply is turned on, the droplets hanging at the tip of the needle are stretched by a combination of electric and magnetic forces. In the space between the tip and the aluminum foil, the droplets are drawn and refined, the solution is volatilized, and the fiber is solidified [54]. Finally, micro-nano fiber membranes with thicknesses of about 2 mm and an average weight of 0.3 g/m^2^ were deposited on the aluminum foil after 30 minutes. In the experiment, we locked the spinning distance to fixed distances of 4, 6, 8, 10, 12, and 14 mm. The critical spinning voltages were then tested at various distances. The magnets were then replaced by different magnetic field strengths (30, 93, and 154 mT) or the spinning solution was changed with different mass ratios of nanoparticle (1, 2 and 3 wt %). For the convenience of subsequent reading, the detailed information of each sample—including the preparation method, composition, mass ratio, and magnetic field strength—has been numbered and listed in Table 1. Then the critical spinning voltages were t tested again at different distances to confirm the effect of the magnetic field strength and mass ratio of nanoparticles to critical spinning voltages at each spinning distance.

Theoretical simulations of electric and magnetic fields in MES were further conducted to confirm the distribution of the electromagnetic field and further ensure the rationality of the experiment. Figure 1b–f shows the simulated electric and magnetic fields. All simulation works were done with COMSOL Metaphysics Finite Elemental Analysis software [46,55]. The modeling uses a metal needle with an outer diameter of 0.72 mm and inner diameter of 0.42 mm placed vertically 10 mm directly above the strip magnet (2.47 × 1.62 × 0.64 cm = 2.56 cm^3^). In the simulations, the needle tip and the permanent magnet were not placed horizontally because we only wanted to confirm how the electrostatic and magnetic fields are distributed between the needle and the collection. In particular, the distribution of the magnetic field directly determines how long the spinning distance should be set in the MES to ensure the magnetic field works. As shown in Figure 1b–f, the electric field distribution around the needle, as shown in equipotential lines and the magnetic field was indicated by the color distributed throughout the entire space with the color bar on the right. Here, we mainly consider the distribution of the magnetic field intensity (*H*, unit A/m) formed by magnets with different magnetic field strengths (*B*, unit mT). The relation between *H* and *B* is calculated by the formula *B* = *μH*, where *μ* is the magnetic susceptibility [55,56].

Because the needle is connected to the positive electrode of the HVDC power supply and the aluminum foil attached to the magnet is grounded, we keep the output voltage of the positive electrode of the HVDC power supply at 2 kV and divide the voltage into 10 equal parts. Then each equal part, illustrated by an equipotential line, represents a voltage drop of 0.2 kV. In the space between the needle and the magnet, the voltage decreases gradually, as shown, as the values on the potential lines gradually decrease. In Figure 1b–d, the magnetic field strength of the magnet is 93 mT, but the spinning distance between the needle tip and the magnet increases from 4 cm (b) to 8 cm (c) and 14 cm (d). It can be seen from Figure 1b–d that the spinning distance greatly influences the electric field. The smaller the spinning distance, the denser the equipotential lines become, which indicates a more concentrated and larger electric field force. The same conclusion can also be found in Figure S1 (the effect of distance on the electric field, where the arrow represents the magnitude of the electric field force) in the supporting information. 

At the same time, the distance between the tip of the needle and the magnet will also affect the magnetic field distribution. As shown in Figure 1b–d, the closer the tip of the needle to the magnet, the greater the magnetic field force. The needle tip may be assimilated at 4 mm, making the magnetic field distribution larger than that at 8 mm. The needle tip is too far away from the magnet at 14 mm, and the magnetic field is more divergent. However, as seen in analysis of the color bar, the magnetic field force changes are not very large, and they are all in the same order of magnitude; therefore, the 8 mm spinning distance is the most suitable for greater concentration. Next, keeping the spinning distance of 8 mm, the magnetic field strength on the surface of the magnet changes from 30 mT (e) to 93 mT (c) and 153 mT (f). As shown in Figure 1c,e,f, the spatial distribution of the magnetic field remains the same, but the energy value of the color bar changes a lot. The same conclusion can also be obtained in Figure S2 (the influence of the spinning distance and the magnetic field strength on the pure magnetic field, where the arrow indicates the magnitude of the magnetic field force) in the supporting information. 

As shown in Figure 1b–f, the magnetic field rapidly decays from the surface to the outsides of the magnet. The effective working distance is about 15 mm, and the change within 8 mm is significant. Therefore, the effective working distance of the magnetic field is very small, which is just suitable for the small distance of near-field spinning. This further confirmed our initial idea of introducing a magnetic field into the near-field electrospinning and proposed the concept of MES, which involves combining the actions of the magnetic and electric fields to further stretch the nanofibers and reduce the spinning voltage.

### 2.3. Characterization

The morphology and structure of the spun fibers were measured and characterized by scanning electron microscope (SEM, Hitachi TM-1000 (Qingdao, China)). All samples were coated with an evaporated gold thin film before SEM imaging to ensure high conductivity. The analysis of the organic component PVP used a Fourier transform infrared spectrometer (FTIR, Thermo Scientific Nicolet iN10 (Qingdao, China)) for characterization. The crystal structure of the fibers was characterized by X-ray diffraction (XRD, Rigaku D/max-2400 (Qingdao, China)). In addition, the magnetic properties of the fibers were measured by the vibrating sample magnetometer (VSM) of a physical properties measurement system (PPMS, Quantum Design) sweeping the external field from −15,000 to 15,000 Oe at 300 K. The mechanical properties of the untreated and treated paper-based relic samples were tested on a computer-controlled tensile testing system (INSTRON 3699, Shanghai Instron Co., Ltd.). The electronic conductivity was measured by an ST2643 ultra-high resistance micro current tester (Suzhou Jingge Electronic Co., Ltd. (Qingdao, China)). The diameter of each test sample was fixed at 0.9 cm. EMI shielding effectiveness was tested by DR-913G fabric anti-EMI radiation performance tester (Wenzhou Darong Textile Instrument Co., Ltd. (Qingdao, China)). 

## 3. Results and Discussion

**Figure 2:**
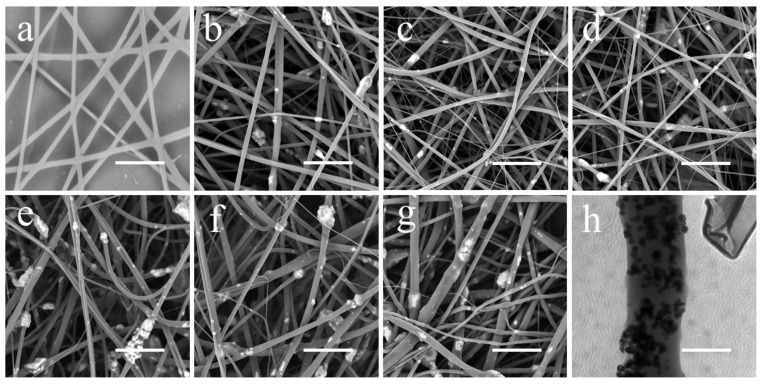
SEM images of (**a**) PVP and (**b**) PVP/γ-Fe_2_O_3_ nanofibers fabricated by near-field electrospinning (NFES) without an auxiliary magnetic field, and (**c**–**g**) PVP/γ-Fe_2_O_3_ nanofibers fabricated by MES; (**c**–**e**) PVP/γ-Fe_2_O_3_ nanofibers with a mass ratio of γ-Fe_2_O_3_ being 1 wt % and different magnetic field strengths; (**c**) 30 mT, (**d**) 93 mT, and (**e**) 154 mT. PVP/γ-Fe_2_O_3_ nanofibers with a magnetic field strength of 93 mT and different mass ratios of γ-Fe_2_O_3_ being (**f**) 2 wt % and (**g**) 3 wt %. (**h**) TEM image of PVP/γ-Fe_2_O_3_ nanofibers fabricated by MES with a magnetic field strength of 93 mT and a mass ratio of 1 wt %. The scale of (**a**–**g**) is 5 μm, and the scale of (**h**) is 600 nm.

**Figure 3:**
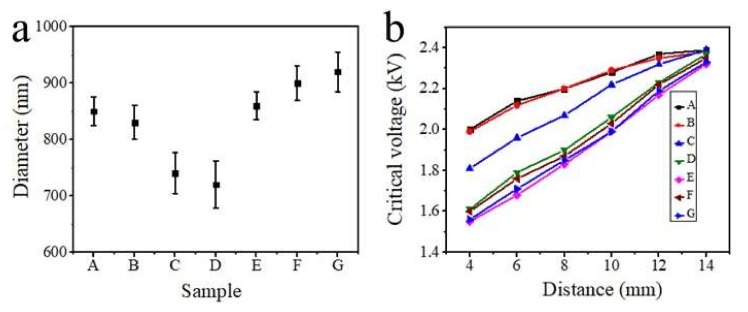
(**a**) Corresponding diameter analysis of Figure 2 and (**b**) the critical voltage of PVP nanofibers fabricated by NFES and PVP/γ-Fe_2_O_3_ nanofibers by MES. The detailed synthetic information of Sample A-G is displayed in Table 1.

First, the pure PVP and PVP/γ-Fe_2_O_3_ nanofibers with a mass ratio (γ-Fe_2_O_3_) of 1 wt.% were prepared by NFES with the spinning voltage and distance fixed at 2.5 kV and 8 mm, respectively. The obtained fibrous morphologies shown in Figure 2a,b indicate that the introduction of γ-Fe_2_O_3_ nanoparticles in the PVP fibrous system does not significantly change the morphology of electrospinning nanofibers but could result in a rough fiber surface. In addition, the diameter of the fibers decreases slightly after the introduction of γ-Fe_2_O_3_ nanoparticles (Figure 3a). Notably, the critical voltages for NFES of pure PVP and PVP/γ-Fe_2_O_3_ nanofibers are similar, suggesting that without an auxiliary magnetic field, the magnetic particles have no significant influence on the NFES process. 

Subsequently, magnetic field strengths varying from 30 mT, 93 mT, to 154 mT were applied for the NFES of PVP/γ-Fe_2_O_3_ nanofibers. As with PVP/γ-Fe_2_O_3_ nanofibers prepared by NFES, PVP/γ-Fe_2_O_3_ nanofibers prepared by MES (a magnetic field-auxiliary NFES method) also show rough fiber surfaces (Figure 2c–e). Furthermore, the corresponding fiber diameter and the critical voltage at each spinning distance are displayed in Figure 3a–b. Obviously, with the introduction of the magnetic field into the NFES process, the fiber decreases; however, when the applied magnetic field reaches a very high strength of 154 mT, the fiber diameter increases, which may be because magnetic field strength that is too high will not leave enough time and space to make the fiber stretch further. In addition, the critical voltage of NFES decreases with the increase of extra magnetic field strength, indicating that extra magnetic field strength could decrease the critical voltage for NFES and lower energy consumption. Taking the above points into consideration, the magnetic field strength is optimized at 93 mT. 

Besides the extra magnetic field, the γ-Fe_2_O_3_ content has been studied here to explore the effect of the γ-Fe_2_O_3_ content on the morphology, fiber diameter, and NFES critical voltage of the PVP/γ-Fe_2_O_3_ nanofibers. With the increase of γ-Fe_2_O_3_ content, *i.e.*, the mass ratio of γ-Fe_2_O_3_ from 1 wt.% for sample D to 2 and 3 wt.% for samples F and G, respectively, the fiber surface becomes rougher (Figure 2d,f,g), and the fiber diameter is significantly increased (Figure 3a), possibly due to the serious aggregation of magnetic γ-Fe_2_O_3_ nanoparticles. In addition, the critical voltage for NFES decreases with the increase of γ-Fe_2_O_3_ content (Figure 3b), possibly because of the increased magnetic field strength caused by the increased γ-Fe_2_O_3_ content. Thus, the optimized γ-Fe_2_O_3_ content should be 1 wt.%. Furthermore, TEM observation has been applied to investigate the morphology of the optimized sample D (Figure 2h). It can be seen that the diameter of a nanofibers is about 700 nm, and no obvious agglomeration of nanoparticles has been observed, indicating that the nanoparticles on the surface and inside of the nanofibers are relatively uniformly dispersed.

**Figure 4:**
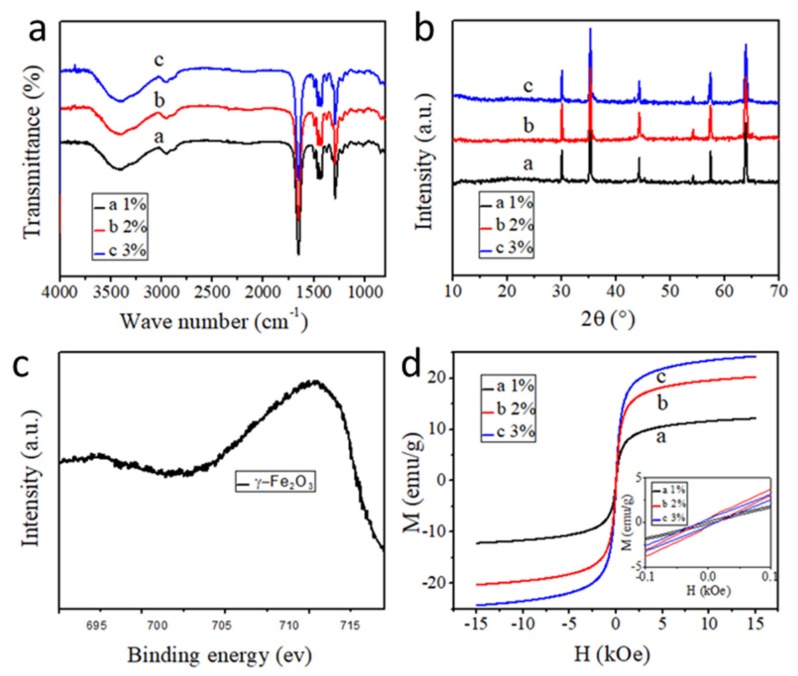
(**a**) FTIR spectra (**b**) XRD patterns, and (**d**) hysteresis loop of PVP/γ-Fe_2_O_3_ nanofibers fabricated by MES with different γ-Fe_2_O_3_ contents. (**c**) High-resolution Fe 2p XPS spectrum of PVP/γ-Fe_2_O_3_ nanofibers.

**Figure 5:**
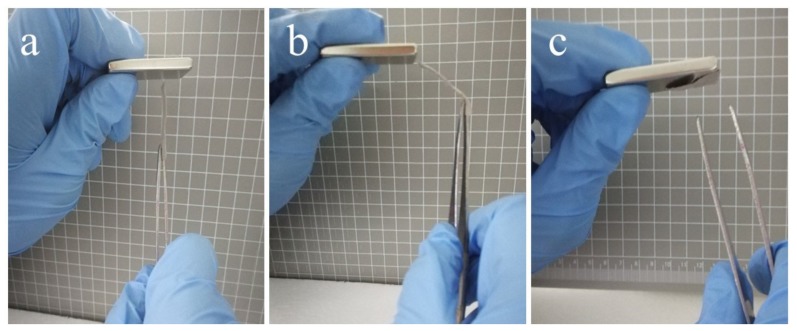
Flexibility and magnetic properties of PVP/γ-Fe_2_O_3_ nanofibers fabricated by MES (**a**) directly under the magnet, (**b**) on the left of the magnet, and (**c**) released from the left of the magnet.

FTIR spectra were first applied to investigate the influence of γ-Fe_2_O_3_ nanoparticles on the organic component PVP. As shown in Figure 4a, all characteristic peaks of PVP could be found in the composite fibers, even when the γ-Fe_2_O_3_ content increases, which confirms that the main components of PVP have not been affected by γ-Fe_2_O_3_ nanoparticles [57]. Further XRD patterns show the characteristic diffraction peaks of γ-Fe_2_O_3_ at 30.7°, 36.1°, 44.7°, 54.8°, 57.6°, and 64.8° (Figure 4b), corresponding to the (220), (311), (400), (422), (511), and (440) crystal planes of γ-Fe_2_O_3_, respectively [1,58,59]. The XPS spectrum of PVP/γ-Fe_2_O_3_ nanofibers shown in Figure 4c further demonstrates the high purity of γ-Fe_2_O_3_ in the composite fibers [60]. The magnetization, M, versus the applied magnetic field, H (from −15 kOe to 15 kOe), and the enlarged image (from −0.3 to 0.3 kOe) are shown in Figure 4d. The hysteresis loop demonstrates the superparamagnetic behavior of the composite nanofibers [47,61,62]. The magnetization increases sharply when the applied field is increased; M is nearly saturated at about 2 kOe, and *M*_s_ is around 25 emu/g for PVP/γ-Fe_2_O_3_ nanofibers fabricated by MES with 3 wt % γ-Fe_2_O_3_, 18 emu/g for 2 wt %, and 10 emu/g for 1 wt %. The coercive force and remnant magnetization are very small (not decreased to zero), which indicates the superparamagnetic behavior of the PVP/γ-Fe_2_O_3_ nanofibers. Additionally, the coercive force increases as the magnetic particles increase, which may be caused by the irresistible γ-Fe_2_O_3_ nanoparticles that are too large or aggregated [26,63], as shown in Figure 2h. As shown in Figure 5a–c, the nanofiber membrane was dragged with a tweezer, moving from directly below the magnet to the right. During this process, the nanofiber membrane obviously received the force from the magnet block because of the magnetic γ-Fe_2_O_3_ nanoparticles. The nanofiber membrane can bend to the left between the tweezer and the magnet under the effect of the magnetic field without breaking, indicating that the nanofiber membrane has good flexibility and mechanical properties. When the tweezer was released, the nanofiber membrane was quickly attracted to the magnet, which further confirmed the magnetic properties and flexibility of the nanofiber membrane. Obviously, the composite fibers possess a good mechanical property, including good flexibility and stretch ability and strong magnetic property, indicating promising potential for flexible magnetic materials.

**Figure 6:**
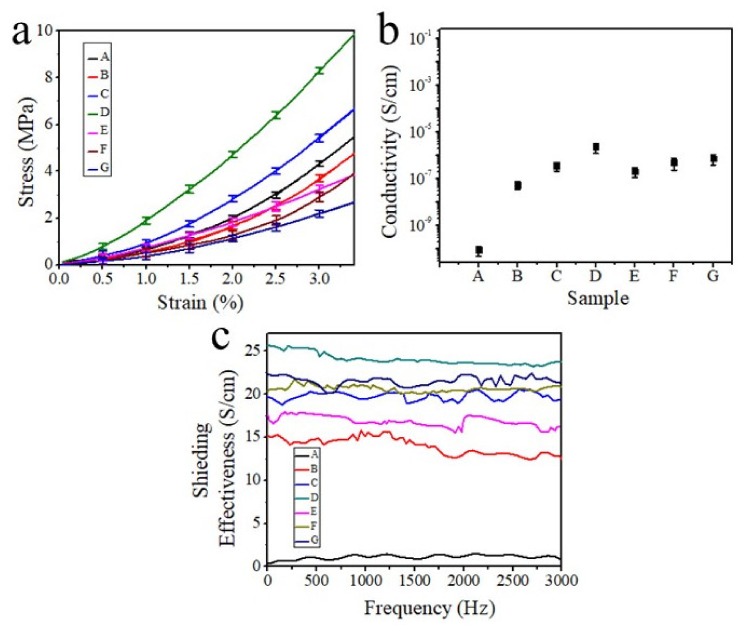
(**a**) Mechanical properties, (**b**) electrical conductivity, and (**c**) electromagnetic shielding performance of the PVP/γ-Fe_2_O_3_ nanofiber membrane fabricated by MES; A: PVP fibers fabricated by NFES; B: PVP/γ-Fe_2_O_3_ nanofibers fabricated by NFES; C–E: PVP/γ-Fe_2_O_3_ nanofibers fabricated by MES with a magnetic field strength of (**c**) 30 mT, (D) 93 mT, and (E) 154 mT; F–G: PVP/γ-Fe_2_O_3_ nanofibers fabricated by MES with different mass ratios (γ-Fe_2_O_3_) of (F) 2 wt % and (G) 3 wt %.

The mechanical properties of the electrospinning nanofibers were investigated and are shown in Figure 6a. The results show that, without the magnetic field, the added γ-Fe_2_O_3_ nanoparticle cannot change the mechanical properties of the B fibers. In contrast, the mechanical properties of the composite nanofibers via MES have been significantly enhanced when magnetic fields with strengths of 30 and 93 mT are introduced, corresponding to C and D fibers, respectively. However, when the magnetic field strength further increased to 154 mT, the mechanical properties of the spun nanofibers were reduced, corresponding to E fibers, possibly due to the increased diameter of the fibers. On the other hand, with the increase of the γ-Fe_2_O_3_ content, the mechanical properties of the electrospinning nanofibers also reduce obviously, corresponding to the F and G fibers. This is consistent with the conclusion in Figure 3a. Generally speaking, the finer the fiber, the more regular the molecular chain arrangement, and the mechanical properties of the fiber will be better [64]. At the same time, the finer the fiber, the larger the specific surface area, and the performance of the loaded substance [65] (for example, electricity, magnetism, fluorescence) will be better. From the comparison of B and D in Figure 6a, loaded magnetic particles can significantly improve the mechanical strength of the fiber. This may be because the in situ magnetic field induces the magnetic particles to be sequentially arranged to avoid demagnetization interactions. The attractive forces between the magnetic particles influence each other, thereby effectively improving the fiber strength. The electrical conductivity of the electrospinning nanofibers was further investigated and is shown in Figure 6b. The thickness of the fiber membranes used for testing was fixed at 2 mm by controlling the spinning time. It was obvious that all composite fibers showed an improved electrical conductivity as compared with pure PVP fibers, corresponding to A fibers. Sample B is more conductive than sample A for the addition of magnetic nanoparticles. The effect of the magnetic field on the magnetic particles in the spun composite fiber changes the fineness and the distribution of the magnetic nanoparticles. As the strength of the magnetic field increases, the fineness of the fiber becomes smaller, and the magnetic particles are more uniformly dispersed, so the conductivity of samples C and D is increased. However, when the magnetic field strength is too large, the composite fibers quickly reach the collection level for the force of the magnetic field to the magnetic particles in the composite fiber. There is not enough time and space for fiber stretching, so the fibers are thick, the particles are agglomerated, and the dispersion is uneven. Therefore, the conductivity of sample E is worse than that of sample D, and the proportion of magnetic particles was increased in samples F and G, while the conductivity was worse. This may be due to the same reason—the agglomeration and uneven distribution of magnetic nanoparticles caused by the large addition ratios. Therefore, the conductivity of samples E, F, and G was not good because of the agglomeration and uneven dispersion of magnetic particles. However, the conductivity of samples F and G is better than that of sample E because the fibers are thicker. In addition, the composite fibers fabricated by MES with a magnetic field strength of 93 mT and a γ-Fe_2_O_3_ content of 1 wt % exhibit the best electrical conductivity, suggesting a promising application for electromagnetic shielding.

According to ASTM D4935 (Standard Test Method for Measuring the EMI Shielding Effectiveness of Planar Materials) and related literature [23], the electromagnetic shielding effectiveness can be defined as:

SE = 10log(*P*_1_/*P*_2_) (1)

where, SE is the logarithmic representation of shielding effectiveness (dB), *P*_1_ is the measured value (power of electromagnetic waves) when no shielding material is placed in the test fixture, and *P*_2_ is the measured value (power of electromagnetic waves) when the tested shielding material is in the test fixture. The electromagnetic shielding properties of the electrospinning nanofibers are shown in Figure 6c. The tested micro-nano fiber membrane has an average thickness of about 2 mm and an average weight of 0.3 g/m^2^. It can be seen that the composite fibers fabricated by MES with a magnetic field strength of 93 mT and γ-Fe_2_O_3_ content of 1 wt % (sample D in the blue line of Figure 6c) exhibit the best electromagnetic shielding performance, which is almost tenfold that of pure PVP fibers. There are some strange “jumps” in some of the curves, which may be due to the unevenness of the device sensitivity and the impact of the agglomerated nanoparticles on the surface of the nanofiber membranes.

## 4. Conclusions

In summary, PVP/γ-Fe_2_O_3_ composite nanofibrous films were prepared by a simple magnetic-electrostatic spinning method. The spinning critical voltage and the fiber diameter of electrospinning nanofibers were obviously reduced in the MES process because of the tensile effect of the magnetic field on the magnetic γ-Fe_2_O_3_ nanoparticles in the PVP/γ-Fe_2_O_3_ composite fibers. In addition, the optimized magnetic field strength and γ-Fe_2_O_3_ content for the synthesis of composite fibers are 93 mT and 1 wt %, respectively. The fibers prepared in these circumstances show the best magnetic property and highest electrical conductivity, which lead to the best EMI shielding performance. 

## Figures and Tables

**Table 1:** Detailed information for each sample.

Sample	Method	Composition	Magnetic Field Strength (mT)	Mass Ratio of γ-Fe_2_O_3_ (wt.%)
A	NFES	PVP	0	0
B	NFES	PVP/γ-Fe_2_O_3_	0	1
C	MES	PVP/γ-Fe_2_O_3_	30	1
D	MES	PVP/γ-Fe_2_O_3_	93	1
E	MES	PVP/γ-Fe_2_O_3_	154	1
F	MES	PVP/γ-Fe_2_O_3_	93	2
G	MES	PVP/γ-Fe_2_O_3_	93	3

## Supplementary Materials

The following are available online at https://www.mdpi.com/2073-4360/12/3/695/s1.
