# Supplementary material for: Magnetic-Electrospinning Synthesis of γ-Fe2O3 Nanoparticle–Embedded Flexible Nanofibrous Films for Electromagnetic Shielding
Source: Polymers (Basel). 2020 Mar 20;12(3):695. doi: 10.3390/polym12030695 (PMC7182905; doi:10.3390/polym12030695)
Supplement: Supplementary file 1 [file polymers-12-00695-s001.pdf]

Supporting Information

for

Magnetic-Electrospinning Synthesis of γ-Fe_2O_3 Nanoparticle–embedded Flexible Nanofibrous Films for Electromagnetic Shielding

Jie Zheng,[1,2,3] Bin Sun,[2*] Xiao-Xiong Wang,[2] Ze-Xing Cai,[3] Xin Ning,[1] Saad M. Alshehri,[4] Tansir Ahamad,[4] Xing-Tao Xu,[3]* Yusuke Yamauchi[5,6] and Yun-Ze Long[2]*

1 Industrial Research Institute of Nonwovens & Technical Textiles, College of Textiles & Clothing, Qingdao University, Qingdao 266071, China

2 Collaborative Innovation Center for Nanomaterials & Devices, College of Physics, Qingdao University, Qingdao 266071, China

3 International Center for Materials Nanoarchitectonics (WPI-MANA) and International Center for Young Scientists (ICYS), National Institute for Materials Science (NIMS), 1-1 Namiki, Tsukuba, Ibaraki 305-0044, Japan

4 Department of Chemistry, College of Science, King Saud University, Riyadh, 11451, Saudi Arabia

5 School of Chemical Engineering and Australian Institute for Bioengineering and Nanotechnology (AIBN), The University of Queensland, Brisbane, QLD 4072, Australia

6 Department of Plant & Environmental New Resources, Kyung Hee University, 1732 Deogyeong-daero, Giheung-gu, Yongin-si, Gyeonggi-do 446-701, South Korea

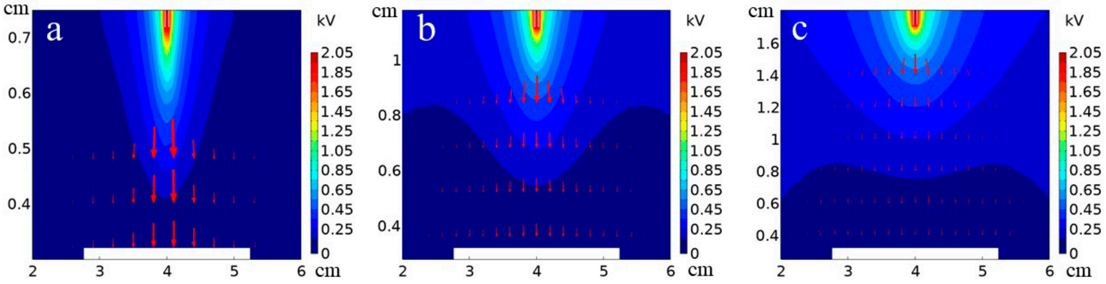

Fig. S1 The simulation of the effect of the spinning distance on the electric field. The arrow represents the magnitude of the electric field force. (a) 4 mm, (b) 8 mm and (c) 14 mm.

Supplementary Note for Fig. S1:

The tip of the needle is connected to the positive pole of an HVDC power supply, with a constant voltage output of 2 kV. The aluminum foil adhered to the magnet is grounded and connected to the negative pole of the same HVDC power supply. The electric field distributions at different spinning distances (distances from the tip to the magnet) were shown in Fig. s1. It can be seen from the Fig. s1 that the smaller the spinning distance, the more concentrated the electric field distribution, and the larger the electric field force (indicated by the arrow with uniform scale). Therefore, the required spinning voltage increases as the spinning distance increases as shown in Fig. 3b.

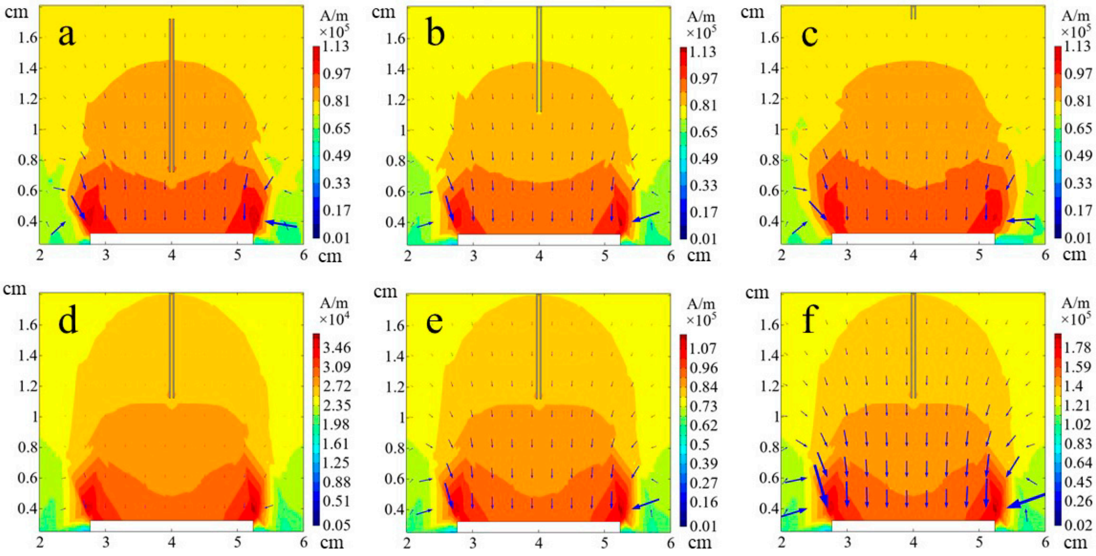

Fig. S2 The simulation of the effect of the spinning distance and magnetic field strength (*B*) on the magnetic field intensity (*H*). (a-c) different distances: (a) 4 mm, 93 mT; (b) 8 mm, 93 mT; and (c) 14 mm, 93 mT. (d-f) Different magnetic field strengths (*B*): (a) 8 mm, 30 mT; (b) 8 mm, 93 mT; and (c) 8 mm, 154 mT. The arrow indicates the magnitude of the magnetic field force.

Supplementary Note for Fig. S2:

The effects of the spinning distance (a-c) and magnetic field strength (B, d-f) on the magnetic field intensity (H) were shown in Fig s2. The arrow indicates the magnitude of the magnetic field force. As shown in the figure, the influence of the spinning distance on the magnetic field distribution is very small. However, the influence of the magnetic field strength on the magnetic field distribution is very large. Excessive stretching will lead to the fiber quickly reaching the collector without sufficient time and space to be fully stretched. Therefore, when the magnetic field strength is too large (for example 154 mT), the fiber will be thicker, and many parallel filaments are shown in Fig. 2e and Fig. 3a in the marked manuscript.
